# Not-So-Novel Michigan Rabbit Calicivirus^1^

**DOI:** 10.3201/eid1608.091803

**Published:** 2010-08

**Authors:** Joana Abrantes, Pedro J. Esteves

**Affiliations:** CIBIO-UP (Centro de Investigação em Biodiversidade e Recursos Genéticos, Vairão–Universidade do Porto, Porto, Portugal (J. Abrantes, P.J. Esteves); Institut National de la Santé et de la Recherche Médicale (INSERM), Unité 892, Université de Nantes, Nantes, France (J. Abrantes); CESPU (Cooperativa de Ensino Superior Politécnico e Universitário), Gandra, Portugal (P.J. Esteves).

**Keywords:** Rabbits, calicivirus, Lagovirus, caliciviridae infections, phylogeny, viruses, letter

**To the Editor:** A disease outbreak in a Michigan rabbitry led Bergin et al. (*1*) to identify a new rabbit calicivirus distinct from rabbit hemorrhagic disease virus, which they designated as Michigan rabbit calicivirus (MRCV). They found that in domestic rabbits from the United States, 2 different forms of rabbit calicivirus with differences in pathogenicity are circulating. Bergin et al. showed that, phylogenetically, MRCV was more closely related to the nonpathogenic rabbit calicivirus (RCV) than to pathogenic strains and used this observation as an argument for its classification as a novel calicivirus. However, they did not include the publicly available sequences of other nonpathogenic strains, such as Ashington (97% of the capsid viral protein [VP] 60) and the newly identified *Lagovirus* spp. RCV-A1 (complete genome) (*2*).

Using the same dataset as Bergin et al. and including these sequences, we performed genetic analyses focusing mainly on the capsid VP60. The lack of information for open reading frame 1 for the nonpathogenic strains led to this option. Independently of the sequences’ length, RCV-A1 was more closely related to the *Lagovirus* spp. European brown hare syndrome virus, here used as an outgroup, and clearly apart from a highly supported primary group that was further subdivided into 2 also highly supported subgroups, 1 composed of pathogenic rabbit hemorrhagic disease virus strains and another encompassing the RCV-like group (RCV, Ashington and Lambay [*2*], and MRCV). Here, only the phylogenetic tree that corresponds to the more complete VP60 sequences is shown (Figure).

**Figure:**
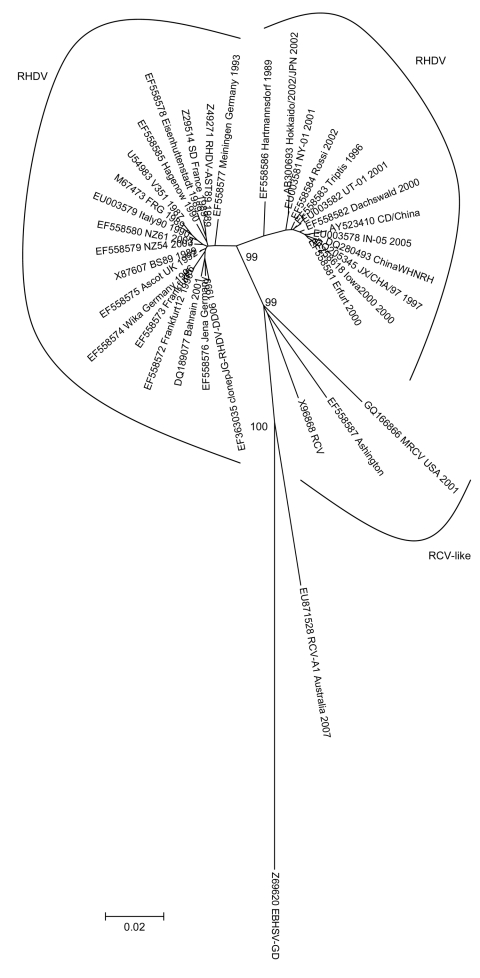
Evolutionary relationships of *Lagovirus* strains. The evolutionary history was inferred by using the neighbor-joining method (*3*) with the pairwise deletion option. The tree is drawn to scale. There were a total of 563 positions (97% of the capsid viral protein [60 aa sequence]). Phylogenetic analyses were conducted in MEGA 4 (*4*). Reliability of the tree was assessed by bootstrap with 1,000 replicates and is indicated in the nodes (only relevant values are shown). Several genetic distance methods were used, and similar results were obtained, but only p-distance is shown. GenBank accession numbers of the sequences used are indicated. Scale bar indicates nucleotide substitutions per site. RDHV, rabbit hemorrhagic disease virus; RCV, rabbit calicivirus; EBHSV, European brown hare syndrome virus.

We conclude that MRCV is not a novel calicivirus but a new variant of the nonpathogenic RCV-like group. However, the low pathogenicity presented by MRCV and the presence of viral RNA in the liver rather than in the intestine are clearly new features among the nonpathogenic RCV-like group (*5*).
