# Supplementary material for: Not-So-Novel Michigan Rabbit Calicivirus
Source: Emerg Infect Dis. 2010 Aug;16(8):1331–2. doi: 10.3201/eid1608.091803 (PMC3298318; doi:10.3201/eid1608.091803)
Supplement: Related texts

# Reply

**DOI:** 10.3201/eid1608.100711

**Authors:** Ingrid L. Bergin, Annabel G. Wise, Steven R. Bolin, Thomas P. Mullaney, Matti Kiupel, Roger K. Maes

**Affiliations:** University of Michigan, Ann Arbor, Michigan, USA (I.L. Bergin); Michigan State University, Lansing, Michigan, USA (A.G. Wise, S.R. Bolin, T.P. Mullaney, M. Kiupel, R.K. Maes)

**In response:** We thank Abrantes and Esteves for their interest in our article (*1*). We demonstrated that a disease outbreak among rabbits in the United States was associated with a calicivirus distinct from members of the species *Rabbit hemorrhagic disease virus* (RHDV) (*2*). Rabbit hemorrhagic disease is classified as a disease of foreign animals in the United States and is caused by the only calicivirus in the genus *Lagovirus* previously associated with disease in rabbits. Our phylogenetic analysis established that Michigan rabbit calicivirus (MRCV) is distinct from RHDV and more closely related to a nonpathogenic rabbit calicivirus (RCV). We are pleased that Abrantes and Esteves agree with us on this point.

In regard to phylogeny, the additional analysis performed by Abrantes and Esteves is an extension to, not an omission of, the original disease-focused paper. It is difficult to understand their objection to the term novel, a point that seems semantic. This term has more than just a phylogenetic connotation, and our use of it is consistent with other reports in this journal (*3**,**4*).

In Abrantes and Esteves’ analysis, although RCV, Ashington, MRCV, and RCV-A1 appear to share common ancestry, MRCV branches separately from Ashington and RCV. The limited sequence availability for RCV and Ashington hampers detailed analysis of the interrelatedness of these viruses.

In conclusion, we describe MRCV as a novel calicivirus on the basis of its identification as the first non-RHDV *Lagovirus* sp. detected in the United States, its unique pathogenic potential to rabbits among the currently described non-RHDV lagoviruses, and its genetic distinction from RHDV. The phylogenetic relationships of the non-RHDV caliciviruses will no doubt be further refined as more members with complete or near-complete sequences, like MRCV, become available. Perhaps this will shed further light on the apparent pathogenicity of MRCV under certain circumstances.

## References

[1] Abrantes J, Esteves PJ. Not-so-novel Michigan rabbit calicivirus [letter]. Emerg Infect Dis. 2010;16:1331–2. 10.3201/1608.10071120678344PMC3298318

[2] Bergin IL, Wise AG, Bolin SR, Mullaney TP, Kiupel M, Maes RK. Novel calicivirus identified in rabbits, Michigan, USA. Emerg Infect Dis. 2009;15:1955–62. 10.3201/eid1512.09083919961675PMC3044539

[3] Hall AJ, Cassiday PK, Bernard KA, Bolt F, Steigerwalt AG, Bixler D, Novel *Corynebacterium diphtheriae* in domestic cats. Emerg Infect Dis. 2010;16:688–91.2035038910.3201/eid1604.091107PMC3321947

[4] Hofmann MA, Renzullo S, Mader M, Chaignat V, Worwa G, Thuer B. Genetic characterization of toggenburg orbivirus, a new bluetongue virus, from goats, Switzerland. Emerg Infect Dis. 2008;14:1855–61. 10.3201/eid1412.08081819046507PMC2634640
